# Prevention of nosocomial infections in critically ill patients with lactoferrin (PREVAIL study): study protocol for a randomized controlled trial

**DOI:** 10.1186/s13063-016-1590-z

**Published:** 2016-09-29

**Authors:** John Muscedere, David Maslove, John Gordon Boyd, Nicole O’Callaghan, Francois Lamontagne, Steven Reynolds, Martin Albert, Rick Hall, Danielle McGolrick, Xuran Jiang, Andrew G. Day

**Affiliations:** 1 Department of Critical Care Medicine, Queen’s University, Kingston, ON Canada; 2 Centre de recherché du CHU de Sherbrooke, Université de Sherbrooke, Sherbrooke, QC Canada; 3 Department of Medicine, University of British Columbia, Vancouver, BC Canada; 4 Centre de Recherche de l’Hôpital du Sacré-Coeur de Montréal, Division of Critical Care Medicine, Critical Care and Medicine Departments, Université de Montréal, Montréal, QC Canada; 5 Department of Critical Care Medicine, Dalhousie University and the Nova Scotia Health Authority, Halifax, NS Canada; 6 Kingston General Hospital, Room 5-411, Angada 4, 76 Stuart Street, Kingston, ON K7L 2 V3 Canada

**Keywords:** Lactoferrin, Intensive care, Critically ill, Antibiotics, Nosocomial infections, Mechanically ventilated patients, Mortality

## Abstract

**Background:**

Nosocomial infections remain an important source of morbidity, mortality, and increased health care costs in hospitalized patients. This is particularly problematic in intensive care units (ICUs) because of increased patient vulnerability due to the underlying severity of illness and increased susceptibility from utilization of invasive therapeutic and monitoring devices. Lactoferrin (LF) and the products of its breakdown have multiple biological effects, which make its utilization of interest for the prevention of nosocomial infections in the critically ill.

**Methods/design:**

This is a phase II randomized, multicenter, double-blinded trial to determine the effect of LF on antibiotic-free days in mechanically ventilated, critically ill, adult patients in the ICU. Eligible, consenting patients will be randomized to receive either LF or placebo. The treating clinician will remain blinded to allocation during the study; blinding will be maintained by using opaque syringes and containers. The primary outcome will be antibiotic-free days, defined as the number of days alive and free of antibiotics 28 days after randomization. Secondary outcomes will include: antibiotic utilization, adjudicated diagnosis of nosocomial infection (longer than 72 h of admission to ICU), hospital and ICU length of stay, change in organ function after randomization, hospital and 90-day mortality, incidence of tracheal colonization, changes in gastrointestinal permeability, and immune function. Outcomes to inform the conduct of a larger definitive trial will also be evaluated, including feasibility as determined by recruitment rates and protocol adherence.

**Discussion:**

The results from this study are expected to provide insight into a potential novel therapeutic use for LF in critically ill adult patients. Further, analysis of study outcomes will inform a future, large-scale phase III randomized controlled trial powered on clinically important outcomes related to the use of LF.

**Trial registration:**

The trial was registered at www.ClinicalTrials.gov on 18 November 2013. Trial registration number: NCT01996579.

**Electronic supplementary material:**

The online version of this article (doi:10.1186/s13063-016-1590-z) contains supplementary material, which is available to authorized users.

## Background

Nosocomial infections (NIs) remain an important cause of morbidity, mortality, and increased health care costs in hospitalized patients [1–3]. This is particularly problematic in intensive care units (ICUs) where NIs occur in 25 to 35 % of ICU patients and account for approximately 25 % of all nosocomial infections in the hospital [4, 5]. In ICU patients, mechanical ventilation is an important risk factor for NIs as more than two thirds of infections originate at three major sites: the respiratory tract, the blood stream, and the urinary tract [6]. Further, since NIs are increasingly caused by multidrug-resistant bacteria, clinicians frequently prescribe broad spectrum antibiotic regimens. In turn, this sets the stage for pseudomembranous colitis (PMC), another potentially lethal NI [7]. The prevention of NIs has the potential to improve patient outcomes and reduce the cost burden of increasingly broad spectrum, prolonged courses of antibiotics administered for suspected or confirmed infections.

Lactoferrin (LF), an 80-kDa, multifunctional glycoprotein of the transferrin family, is distributed widely in humans particularly in secretions of exocrine glands and specific neutrophil granules. The highest concentrations of LF are found in breast milk and colostrum. As an important component of the human innate immune system, it has many appealing properties that may prove effective for the prevention of NIs [8]. LF has the ability to bind iron, an important element for microbial growth, thereby reducing its availability to microorganisms and making it bacteriostatic [9–11]. In addition, LF has bactericidal effects on microorganisms attributable to its highly cationic charged terminus, which binds to bacterial surfaces [12]. The binding of LF to the bacterial surface destabilizes the bacterial outer membrane, thus enhancing bacterial susceptibility to osmotic shock, lysozyme, or other antimicrobial molecules [13–15]. This antibacterial activity has been documented against many important human pathogens, including: *Escherichia coli*, *Staphylococcus aureus*, *Klebsiella* sp*., Acinetobacter* sp., *Pseudomonas* sp., *Salmonella* sp., and *Proteus* sp. [16–18]. Additionally, LF has been shown to exhibit activity against *Candida albicans* and *Candida krusei* and to inhibit the formation of biofilms by *Pseudomonas aeruginosa* and oral bacteria [19–21]. This may be an important attribute of LF as biofilm formation is a large contributor to device-related infections [22].

Other reported effects of LF include its ability to neutralize lipopolysaccharide (LPS) and promote the growth of beneficial bacteria in the GI tract including *Bifidobacteria* [23, 24]. Lastly, LF has the ability to modulate the immune system including increasing the size of Peyer’s patches, increasing serum immunoglobulin levels, decreasing inflammatory cytokines and increasing anti-inflammatory cytokines such as interleukin-10 (IL-10) [25]. In healthy human volunteers, the administration of bovine lactoferrin (bLF) was associated with increased T-cell activation (total, helper, and cytotoxic) and increased antioxidant status [26]. Although LF is a polypeptide, it is resistant to digestion in the digestive tract with 60 to 80 % of bovine LF exiting the stomach intact [27]. Biologically active peptides survive after transit through the small intestine with antibacterial activity remaining similar or increased compared to intact LF [28–30].

The properties of LF make it a potential tool for the prevention of NIs in critically ill patients since its enteral administration may reduce the overgrowth of pathogens, which is an important cause of NIs in the critically ill [31]. We hypothesize that the administration of LF per os and via the nasogastric route to critically ill mechanically ventilated patients will reduce NIs, reduce antibiotic usage, and result in improved patient outcomes and survival. To test our hypothesis, we will first conduct a phase II randomized control trial (RCT) of LF for prevention of NIs with antibiotic-free days as the primary outcome supported by biomarker and mechanistic data. The data from this study will inform whether to proceed to a larger definitive study. Herein we report the protocol for the phase II study which is written in accordance with standardized reporting guidance from SPIRIT (see Additional file 1).

## Methods/design

**Fig. 1:**
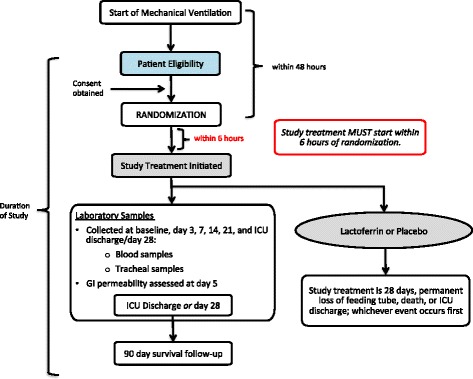
An overview of the study process

A phase II randomized, multicenter, double-blinded trial in five Canadian tertiary ICUs. Study sites include: Kingston General Hospital (Kingston, ON), Hôpital du Sacré-Coeur de Montréal (Montreal, QC), Sherbrooke (Sherbrooke, QC), Royal Columbian Hospital (New Westminster, BC), and Ottawa Hospital (Ottawa, ON). An overview of the study process is provided in Fig. 1.

### Study population

Adult mechanically ventilated patients in the ICU meeting all of the inclusion criteria and none of the exclusion criteria.

#### Inclusion criteria

1. Adult patients (aged 18 years or older)
2. Invasive mechanical ventilation for 48 h or less
3. Patient is expected to be mechanically ventilated for longer than 72 h

#### Exclusion criteria

1. Patient is expected to be in ICU for less than 72 h from the time of randomization (due to imminent death, withdrawal of life sustaining therapies, or discharge)
2. Presence of a contraindication to enteral feeding
3. Lack of access to the oral cavity
4. An allergy or sensitivity to LF or bovine-derived proteins or milk
5. Patient is immunocompromised (post organ transplantation, acquired immunodeficiency syndrome (AIDS), neutropenia (fewer than 1000/cc absolute neutrophils), corticosteroids (more than 20 mgs/day of prednisone or equivalent for more than 6 months))
6. Fulminant liver failure or end stage liver disease (Child’s class C)
7. Life expectancy less than 6 months due to preexisting conditions
8. Patient is pregnant or lactating
9. Enrollment in an industry-sponsored interventional trial (coenrollment in other interventional studies allowed if there is no interaction between the interventions)
10. Patient has undergone prior randomization in this study

### Study intervention

Patients will be randomized to receive LF or placebo. Patients randomized to the LF arm will receive LF solutions delivered to the oral cavity as a mouth swab and LF down a nasogastric tube. The LF used for the study will be bLF, supplied by Advanced Orthomolecular Research [32]. The dosage of bLF will be a total of 2 g administered in four divided doses per day which is similar to that used in other human studies. Following enrollment, the study intervention will start within 6 h of randomization. Study treatment will be discontinued after 28 days from the time of randomization, permanent removal of the feeding tube, or death, whichever occurs first. All patients will receive standard ICU care for critically ill mechanically ventilated patients. The prescription of antibiotics will be left to the treating team.

### Random allocation

A password-protected web-based central randomization system is used to allocate patients to study treatments such that investigators, research staff and patients are blinded to the next allocation and an audit trial is maintained. Clinicians will remain blinded during the study, which will be maintained by administering LF or placebo in opaque syringes such that the placebo and active solutions are indistinguishable from each other. Randomization will be stratified by site using permuted blocks of variable size. The randomization list will be computer-generated by the trial biostatistician using SAS (SAS Inc., Cary, NC, USA) and securely stored by the study biostatistician and the IT manager of the central randomization system.

### Follow-up

Patients will be followed daily until day 28. Mortality will be determined for the ICU stay, hospital stay, and at 90 days. All patients will have blood samples drawn for biomarkers (C-reactive protein (CRP), procalcitonin (PCT), and IL-6), as well as for genome-wide expression profiling, at baseline and on days 3, 7, 14, 21, and 28. Cytokines will be measured using multiplex enzyme-linked immunosorbent assays (ELISAs), with samples prepared on site and shipped to a central laboratory for processing. Blood for gene expression analysis will be collected in PAXgene tubes, frozen according to the manufacturer’s protocol, and shipped to a central genomics facility for ribonucleic acid (RNA) extraction and expression profiling using the Affymetrix PrimeView microarray [33, 34]. Tracheal samples for bacterial cultures will be obtained at baseline and on days 3, 7, 14, 21, and 28. In addition, given the nature of the intervention, no specific provisions have been made for ancillary or post-trial care of participants.

### Adverse events

All patients will be monitored closely for the development of serious adverse events, and reported as per Good Clinical Practice [35], Health Canada [36] and local ethics’ requirements. For serious adverse events, a form on the electronic Case Report Form (eCRF) is completed by local research personnel to capture the pertinent data.

### Protocol violations

A protocol violation is defined as noncompliance with the study protocol and/or procedures. For protocol violations, local research personnel are required to complete a protocol violation report that is reported to the study leader.

### Data management

Enrolled patients are assigned a numeric identifier to maintain confidentiality and no personal identifiers are collected. All study data is entered into an eCRF (REDCap) [37], which is housed on a secure server at Queen’s University, Kingston, ON. Paper-based study material is stored in a secure location, with access limited to study personnel.

### Primary outcomes

The primary outcome for this phase II study will be antibiotic-free days, defined as being alive and free of antibiotics 28 days after randomization. This measure is similar to ventilator-free days, which is widely used in the critical care literature and incorporates antibiotic status, ICU status, and mortality in the 28 days from study enrollment [38].

### Secondary outcomes

1. *Feasibility (recruitment rates*, *protocol adherence*, *data completeness*, *and time required to capture data)*: the goal for the recruitment rate will be an average of 0.75 patients per week/per site. Protocol adherence will be considered successful if more than 80 % protocol adherence is achieved. Evaluation of protocol adherence will include administration of study medications, obtaining biological samples and carrying out laboratory testing protocols. The goal for data completeness is 100 %, but success will be defined at knowing the status of more than 95 % of the 28-day outcomes. Lastly, the time needed to capture required data will be used to determine the budget and feasibility of a future definitive RCT
2. *Clinical outcomes*: antibiotic utilization will be measured by the prescription of new antibiotics after 72 h of admission to ICU, and number of defined daily doses (DDD) of antibiotics. Blinded central adjudication of nosocomial infection (longer than 72 h from admission to ICU) will be determined using standardized definitions and procedures. Change in organ function post randomization will be measured using the Sequential Organ Failure Assessment (SOFA) score [39]. We will also record hospital and ICU length of stay, and hospital survival and 90-day mortality
3. *Laboratory and biochemical outcomes*: the effects of LF will be evaluated using biomarker analysis, i.e., measurement of levels of sequential PCT, CRP, and IL-6 at baseline, day 3, day 7 and then weekly until 28 days post randomization. Gastrointestinal permeability will be measured using the Lactulose/Mannitol Ratio at day 5 [40, 41]. Immune function will be evaluated based on the production of tumor necrosis factor alpha (TNF-α) in response to an LPS stimulation assay [42]. Rates of tracheal colonization will be determined by the number of tracheal samples positive for potential pathogens
4. *Genomics substudy*: the biological mechanisms of LF action will be explored using gene expression profiling. In a subset of patients (continuously recruited from one study site), an additional tube of blood will be collected at the abovementioned time points for RNA extraction and gene expression profiling. Bioinformatic analyses will examine differences in gene expression at baseline between control and treatment groups, changes in the treatment group in response to therapy, and differences between patients with and without the primary outcome

### Statistical analysis

#### Sample size

From a study that enrolled a similar population, the mean and standard deviation of antibiotic-free days was: mean (SD), 14 (9) [43]. A 25 % increase in antibiotic-free days would be clinically significant and feasible if LF has the expected biological activity. To detect a 25 % increase in antibiotic-free days to 17.5 days in the intervention arm with 80 % power at a two-sided alpha of 0.05, a sample size of 210 (105 subjects per arm) is required. We believe that this sample size will be adequate to reliably assess all feasibility and laboratory outcomes but may not be powered for all clinical outcomes.

#### Analysis of primary and secondary outcomes

The main analysis will provide a histogram showing the distribution of the antibiotic-free days per arm. Although the antibiotic-free days will not be normally distributed, the sample is large enough that the mean will be approximately normally distributed, so the mean difference with corresponding 95 % confidence intervals and *p* values will be estimated by an analysis of variance blocking by site. A sensitivity analysis will use an exact permutation test stratified by site to confirm the main conclusion. For transparency we will report both the primary and sensitivity analysis.

Feasibility outcomes will not be compared by arm but will be provided overall using descriptive statistics (recruitment rates, adherence and completeness proportions and mean time needed to capture required data). Since there are multiple clinical outcomes that may have low power, we will avoid formal hypothesis testing and focus on presenting the between-arm difference in means or proportions with 95 % confidence intervals. All clinical analyses will control for site.

The distribution of the laboratory and biochemical outcomes will be depicted by arm over time using clustered boxplots. Statistical significance between arms at each time point will be tested by the Wilcoxon-rank sum test, but the multiplicity of tests and clinical important of differences will be considered when interpreting statistical significance. All analysis will adhere to the intent-to-treat principle. We expect the amount of missing data to be trivial (less than 5 %), but details of any missing data will be reported.

#### Subgroup and interim analysis

Due to conflicting results on the effects of LF when used as a treatment for sepsis [44, 45] a priori, we will conduct a subgroup analysis of patients presenting with sepsis [46]. No formal interim analyses are planned.

### Dissemination

#### Protocol amendments

Protocol amendments are communicated to the Research Ethics Boards (REBs)/Institutional Review Boards, project sites, and regulatory agencies. The primary means of communication is via email for study management. Any issues requiring acute attention, or of great significance would be managed via teleconference.

#### Dissemination policy

The authors intend to communicate the trial results via a published manuscript and abstract; however, public access to the participant-level dataset and statistical code will not be granted.

## Discussion

There is extensive medical literature on the prevention of nosocomial infections and multiple clinical practice guidelines for their prevention have been published [47–49]. Although rates of nosocomial infections have decreased in recent years, they continue to occur since present preventive measures are only partially effective [50]. New techniques to reduce the occurrence of nosocomial infections continue to be required and LF has many properties that may make it an ideal agent for this. If the results of this phase II study suggest potential efficacy of LF for the prevention of NIs in mechanically ventilated patients, it will inform the conduct of a larger definitive study. This program of research has the potential to change practice and may improve the morbidity and mortality of critically ill patients.

The human clinical studies of LF that have been reported are mainly observational, although randomized trials have been reported. The majority of RCTs with LF have been on the treatment of *Helicobacter pylori*, these reporting on LF in neonates to either prevent sepsis or nosocomial infections and as treatment for sepsis in adults. A meta-analysis reporting on nine RCTs enrolling 1343 patients on the effect of adding LF to standard therapy found that LF increased the rate of *Helicobacter pylori* eradication (odds ratio (OR) = 2.26, 95 % CI 1.70 to 3.00) and reduced the rate of adverse effects (OR = 0.15, 95 % CI 0.04 to 0.54) [51]. One RCT reported on the effect of bLF administered to very-low-birth-weight neonates; the administration of bLF was associated with a reduced risk of sepsis (risk ratio 0.34, 95 % CI 0.17 to 0.70, *p* = 0.002) [52]. A trial for the prevention of ventilator-associated pneumonia (VAP) in critically ill neonates recently reported that there were no local side effects of the orally administered treatment containing LF, and a lower, but not statistically significant rate of VAP was found in the treatment group (9/1000 ventilator-days versus 17/1000 ventilator-days in placebo group, respectively; *p* = 0.16) [53].

There have been two completed trials of human recombinant lactoferrin (hLF) for the treatment of sepsis. In the first trial of hLF for the treatment of severe sepsis in190 patients, the administration of hLF was associated with a 12.5 % reduction in 28-day all-cause mortality (26.9 % versus 14.4 %, *p* = 0.05) [44, 54]. The reduction in mortality was maintained at 3 and 6 months [55]. A second trial utilized similar enrollment criteria and treatment in randomizing 205 patients. The 28-day all-cause mortality in the hLF arm was 25 % compared to 18 % in the placebo group (*p* = 0.11), although ICU and long-term mortality were increased in the hLF group [45]. However, on combining the results of the two trials in a meta-analysis, the relative risk of hospital mortality was 0.88 (95 % CI 0.35 to 2.26) (unpublished data).The cause for the discrepant results between these studies is unknown.

In summary, although LF has many biological properties which may be beneficial in critically ill patients, investigations thus far have yielded conflicting results. A potential use of LF is for the prevention of NIs and this trial is designed to yield preliminary results in this regard to guide future investigations.

### Trial status

The study began recruiting patients in Kingston, ON in November 2013, and expanded to the additional tertiary sites in September 2014. Recruitment is estimated to continue until July 2016. The final report will be prepared for 2016.

## Additional files

Additional file 1: SPIRIT checklist. (DOC 120 kb) Additional file 2: PREVAIL Protocol. (DOCX 15 kb) Additional file 3: PREVAIL study. (PDF 297 kb)

## Abbreviations

AIDS: acquired immunodeficiency syndrome
bLF: Bovine lactoferrin
CRP: C-reactive protein
DDD: Defined daily dose
hLF: Human lactoferrin
ICU: Intensive care unit
IL: Interleukin
KGH: Kingston General Hospital
LF: Lactoferrin
OR: Odds ratio
PCT: Procalcitonin
RCT: Randomized controlled trial
REB: Research Ethics Board
SD: Significant difference
SOFA: Sequential Organ Failure Assessment
TNF-α: Tumor necrosis factor alpha
VAP: Ventilator-associated pneumonia
