# Supplementary material for: Prevention of nosocomial infections in critically ill patients with lactoferrin (PREVAIL study): study protocol for a randomized controlled trial
Source: Trials. 2016 Sep 29;17:474. doi: 10.1186/s13063-016-1590-z (PMC5041570; doi:10.1186/s13063-016-1590-z)
Supplement: Additional file 2: — PREVAIL Protocol. (DOCX 15 kb) [file 13063_2016_1590_MOESM2_ESM.docx]

**Additional file 2.**

The Research Ethics Boards that approved this study at each of the centers involved are as follows:

1. Kingston General Hospital: Queen’s University and Affiliated Teaching Hospitals Research Ethics Board
2. Ottawa Hospital Corporation: Ottawa Health Science Network Research Ethics Board/Conseil d’éthique de la recherche du Réseau de science de la santé d’Ottawa
3. Royal Columbian Hospital: Royal Columbia-Fraser Health Research Ethics Board
4. Hôpital du Sacré-Coeur de Montréal: Comité d’éthique de la recherche de l’HSCM
5. Sherbrooke: Comité d’éthique de la recherche sur l’humain du Centre Hospitalier Universitaire de Sherbrooke
