# Supplementary material for: Prevention of nosocomial infections in critically ill patients with lactoferrin (PREVAIL study): study protocol for a randomized controlled trial
Source: Trials. 2016 Sep 29;17:474. doi: 10.1186/s13063-016-1590-z (PMC5041570; doi:10.1186/s13063-016-1590-z)
Supplement: Additional file 3: — PREVAIL study. (PDF 297 kb) [file 13063_2016_1590_MOESM3_ESM.pdf]

KINGSTON GENERAL HOSPITAL

76 STUART STREET
KINGSTON, ONTARIO
K7L 2V7
TEL: 613-548-3232

Prevention of Nosocomial Infections in Critically Ill patients with Lactoferrin: PREVAIL Study

INVESTIGATOR:	Dr. John Muscedere
TELEPHONE:	613-549-6666 ext 4642
STUDY SITE LOCATION:	Kingston General Hospital<br>76 Stuart St., Kingston, ON.,<br>K7L 2V7

INTRODUCTION:

You are being invited to allow your family member or person for whom you are the substitute decision maker (who will also be referred to as the "participant") to take part in a research study that will last approximately 3 months. At least 210 patients will participate in this Phase 2 study. It is important that you read and understand the following principles that apply to all study participants/substitute decision makers:

1) Taking part in the study is voluntary
2) Personal benefit to the participant, such as a reduction in the risk of developing a hospital acquired infection, may or may not result from taking part in the study. Knowledge may be gained from this study that may benefit patients in the future.
3) You may withdraw the participant or the participant may withdraw from the study at any time without penalty or loss of any benefits.

The nature of the study, the benefits, risks, discomforts and other information about the study are discussed below. There may be information in this consent form that you do not understand. Any questions you have should be discussed with the staff members. They will explain it to you.

THE NATURE AND PURPOSE OF THIS STUDY:

Your family member or person for whom you are consenting has been admitted to the Intensive Care Unit and is receiving assistance with his/her breathing through the help of a mechanical ventilator. Hospital-acquired infections (sometimes called nosocomial infections) are common in severally ill patients. In these patients, there are many reasons for the increased risk of infections such as decreased immune function and the necessary utilization of devices, catheters and antibiotics in critically ill patients. There are measures that can reduce this risk and these will be used in the care of this patient whether or not you choose to participate in this study. However, current preventive measures are only partially effective and there is a need to study and use new measures for the prevention of hospital-acquired infections.

Lactoferrin is a molecule and has many properties that could make it the ideal agent for the prevention of hospital-acquired infections. Lactoferrin has antibacterial properties (is able to kill or stop the growth of disease causing bacteria and fungal organisms), it improves immune function, and can increase the growth of beneficial bacteria in the bowel. Previous studies, both with animal and human volunteers have shown that lactoferrin is both an anti-infective and anti-inflammatory agent. Lactoferrin has been approved by Health Canada as an antioxidant Natural Health product for the maintenance of good health and is sold in health food stores as a health food supplement. However, Lactoferrin has not been studied for the prevention of infections in critically ill patients and the aim of this study is to determine how well it might do so.

The Lactoferrin that we will be using in this study is extracted from cow's milk, where it is normally naturally present. Lactoferrin derived from cow's milk has similar properties as the Lactoferrin normally produced in the human body and has the same antibacterial, antifungal, immune system enhancing properties and beneficial effects on bacteria in the bowel as discussed in the section above.

This study is being conducted to determine how well a solution of Lactoferrin helps to prevent infections and inflammation in critically ill patients **in addition** to usual care and other measures that are known to be partially effective for the prevention of infections.

If you consent for your family member, or person for whom you are the substitute decision maker, they will be randomly (like the toss of a coin) assigned to one of the two treatment groups with an equal chance of belonging to either group:

- Usual care plus Lactoferrin
- Usual care plus matching placebo

Placebo is an inactive "dummy" treatment which looks like the study drug but contains no active ingredient.

EXPLANATION OF STUDY PROCEDURES TO BE FOLLOWED:

In addition to blood, and other samples that are taken for routine care, once the consent form is signed, and before your family member or the person for whom you are consenting begins receiving study drug, approximately 20-30 mL (about one to two tablespoons) of blood will be drawn for lab tests to measure levels of inflammation in the blood. In addition, bacterial cultures will be done on sputum samples. These cultures and blood samples will be repeated periodically during the course of the study (for a total of up to 40 to 110 mL, or approximately 3-7 tablespoon(s) of blood). If the participant is a woman of childbearing potential, that is a woman who is less than 55 years of age, and has had a menstrual period within the last year, or whose menstrual history is unknown and has not had a hysterectomy, a blood or urine sample will also be obtained for a pregnancy test.

Taking the Study Drug:

Participants being enrolled in this study must begin study treatment within 48 hours of ICU admission. The participant will start receiving the study drug or placebo as soon as possible after the

consent form is signed. Neither you nor your study doctor will be able to decide which group the participant is placed in. The participant will have an equal (50:50) chance of being in the group that will get lactoferrin in addition to usual care and will have an equal (50:50) chance of being in the group that will get placebo in addition to usual care. Neither you nor those taking care of your family member or person that you are consenting for will know which group they have been put in. However, in an emergency this can be found out very quickly, if needed. This type of study is called "double-blind."

The participant will receive the study drug through a tube inserted into the stomach for feeding, and also during routine mouth care. Feeding tubes in patients who are on mechanical ventilators are part of usual care and the tube placed by the team caring for the participant will be used to deliver the Lactoferrin solution to the stomach. The study drug will also be delivered during routine mouth care by soaking a mouth swab in a solution of the Lactoferrin and thoroughly wiping the inside of mouth and teeth with it. The participant will receive the study drug 4 times a day for up to 28 days, removal of the feeding tube, or ICU discharge, whichever event comes first.

Duration of Participation:
The participant's participation will last approximately 3 months. This will include receiving the study drug for up to 28 days, until the feeding tube is removed or until they are discharged from the ICU. Participants will also be contacted by telephone at 3 months after they receive the first dose of study drug to determine their overall health status.

During the participant's duration, they will be monitored for the development of infections and antibiotic use. The treatment of infections will be the responsibility and at the discretion of the team caring for the participant. The participant will be monitored for the development of any adverse effects throughout their period of participation.

POSSIBLE RISKS AND DISCOMFORTS:

It is possible that the study drug will not help your family member or person for whom you are consenting. Currently, no side effects have been reported with the use of Lactoferrin in human participants, but there may be side effects associated with Lactoferrin that we do not know about. Participants may experience some temporary discomfort, bruising, bleeding and in rare instances an infection in the process of drawing blood samples, however this is unlikely, as blood will be drawn from catheters or tubes that are already in place while the participant is in the ICU.
Studies on the safety of Lactoferrin for the unborn child have not been performed. Therefore, patients who are pregnant will not be allowed to participate in this study. If your family member or person for whom you are the substitute decision maker is a woman of childbearing potential, that is a woman who is less than 55 years of age, and has had a menstrual period in the last year, or whose menstrual history is unknown and has not had a hysterectomy, they will be tested for pregnancy at the beginning of the study. During this study, participants will receive care that is standard or usual for their condition.

ALTERNATIVES TO PARTICIPATION:

If you decide not to allow your family member or person for whom you are consenting to take part in this study, it will not affect their medical treatment in any way.

POSSIBLE BENEFITS:

It is possible that Lactoferrin may help prevent hospital-acquired infections. It is also possible that Lactoferrin will not provide any additional benefit. However, the knowledge obtained from this research may help health care professional's better treat other patients undergoing treatment for similar conditions.

COMPENSATION FOR PARTICIPATION:

There is no cost for participation in this research study. The study drug will be provided free of charge. Associated tests related to the study will also be provided at no cost. Neither you nor the participant will be paid for participating in this study.

CONFIDENTIALITY AND RELEASE OF MEDICAL RECORDS:

The participant has the right to privacy, and all information obtained in this study that can identify the participants individually will remain confidential. Only the study participant number will be recorded on study documents and reports. Your family member or person for whom you are giving consent will **not** be revealed in any reports or publications resulting from this study. Governmental and institutional regulatory authorities may inspect and copy the records pertaining to this study. The results of the study may be reported to governmental agencies.

In the rare instances for which regulatory authorities may require participant names, the regulatory authorities will treat such information as confidential, but disclosure to third parties may be required on rare occasions. Therefore, absolute protection of confidentiality by regulatory authorities cannot be promised or implied. Blood samples will be labeled and stored at Kingston General Hospital for up to 25 years after the completion of the study. You or the participant has the right to require that all previously retained identifiable samples are destroyed to prevent future analysis.

VOLUNTARY PARTICIPATION AND WITHDRAWAL:

The participation of your family member or person for whom you are giving consent in this study is voluntary and they or you as the substitute decision maker are free to withdraw from the study at anytime. Refusal to participate or withdrawal will involve no penalties or loss of benefits to which the participant is otherwise entitled, and will not affect future medical care.

OFFER TO ANSWER QUESTIONS ABOUT THIS STUDY:

If you or the participant has questions about this study, the medical condition and/or treatment, or if your family member or person for whom you are consenting experiences a research-related injury or illness, please contact **Dr. John Muscedere at: 613-549-6666 ext 4642.**

Should you be unable to reach the study doctor, please feel free to contact the research coordinator at: **Susan Fleury at 613-549-6666 ext. 3191** or **Nicole O'Callaghan at 613-549-6666 ext. 3793**.

If there are any further questions or concerns not addressed by the research coordinators or principal investigator, please contact **Dr. John Drover**, the Chair of the Critical Care Program, at **613-549-6666 ext. 6335**.

REVIEW AND APPROVAL OF THIS STUDY:

This study has been reviewed and was approved by the Queen's University Health Sciences and Affiliated Teaching Hospitals Research Ethics Board on April 25, 2013 (DMED-1569-13)

If you have any questions about your or your family member's right as a research participant, please contact Dr. Albert Clark, Chair of the Queen's University Health Sciences and Affiliated Teaching Hospitals Research Ethics Board at 613-533-6081.

CONSENT TO PARTICIPATE IN THIS STUDY:

Your consent to allow your family member or the person for whom you are the substitute decision maker to participate in this research should be voluntary and informed. By signing this form, you acknowledge that you have read this information (or had it read to you), have been given an opportunity to ask questions about the information provided in this form, and understand the potential risks and benefits. By signing this form you indicate that you wish to allow the participant to participate in this study at this time.
Having consented, you still have the right to withdraw at any time without jeopardy to the participant. If you wish to withdraw, you should notify the study doctor or study coordinator; you do not have to give a reason if you do not wish to. The participant will not lose any legal rights by your signing this form. You will be given a signed copy of this form to keep and refer to as needed.

The substitute decision maker:

You confirm that this consent represents the participant's presumed will and that you understand it may be withdrawn at any time without detriment to the participant. You know of no previous refusal by the participant to consent before the onset of their incapacity.

Investigator and/or person explaining consent:

You confirm that you will provide, at the time of signing, the participant/substitute decision maker with a copy of the signed consent form and information sheet.
You will consider and respect any explicit wish of a participant/substitute decision maker who does not wish to participate or who wishes to be withdrawn at any time, even if consent has previously been given by the participant/substitute decision maker

Name of Study Participant: ___________________

____ ________________

Printed Name/Signature of Substitute Decision Maker | Date

____ ______________

Time

____ _____________

Signature of Person Explaining Informed Consent | Date

____ _____________

Signature of the Investigator or Designee | Date

CONSENT FOR CONTINUED RESEARCH PARTICIPATION

Participant:

You understand that you are currently participating in a research study. You further understand that the consent for your participation in this research study was initially obtained from your legal representative as you were incapacitated and unable to provide direct consent at the time that this initial consent was requested. You have now recovered to the point where it is felt that you are able to provide direct consent for continued participation in this research study.

The above information has been explained to you and all of your current questions have been answered. You understand that you are encouraged to ask questions, voice concerns or complaints about any aspect of this research study during the course of this study, and that such future questions, concerns or complaints will be answered by a qualified individual or by the investigator(s) listed within this consent document at the telephone number(s) given.

By signing this form you agree to continue to participate in this research study.
A full copy of this information sheet and informed consent form will be given to you.

____ ________________
Signature of Study Participant Date/ Time

____

By signing this consent form you do not waive any legal rights which you otherwise would have been entitled to as a participant in a research study.

____ ______________
Signature of Person Explaining Informed Consent Date

CONSENT FOR ADDITIONAL TESTING:

With your permission, we would like to keep the remaining samples of blood that were obtained for this study for DNA and RNA analysis to try to better understand how lactoferrin/immune response may work. DNA is a molecule in your cells which carries the instructions for inherited characteristics. RNA is a molecule that transmits information from the DNA and controls certain chemical processes in the cell. Plasma is the part of the blood from which the blood cells have been removed. With your permission, we would like to store any remaining blood samples for use in future research related to how lactoferrin and /or immune response may work.
This will not involve any additional blood samples; we would like to keep any blood that remains from the sampling that was completed for study purposes.

Blood samples will be labeled and stored at a determined laboratory facility, for up to 25 years after the completion of the study. Blood samples will not be identified using names or other personal confidential information, only by the study participant number.

If you are a First Nations person, or an indigenous person who has contact with spiritual elders you may want to talk with them before you proceed with the part of the study.
You do not have to agree to this part in order to be in the study, and the decision will not affect the care any participants' receives from the study doctors.

Please pick one of the choices below:

□ Blood taken may be kept and used for the testing of DNA, RNA and plasma for research specifically related to lactoferrin/immune response research.

□ Blood may **NOT** be taken and used for testing of DNA, RNA and plasma for research specifically related to lactoferrin/immune response.

You will be given a signed copy of this form to keep and refer to as needed.

____ ____________
Signature of Study Participant Date

____ ____________
Printed Name of Study Participant Time

____ ____________
Signature of Person Explaining Informed Consent Date

____ ____________
Signature of the Investigator or Designee Date
